# Engineering characterisation of epoxidized natural rubber-modified hot-mix asphalt

**DOI:** 10.1371/journal.pone.0171648

**Published:** 2017-02-09

**Authors:** Ramez A. Al-Mansob, Amiruddin Ismail, Nur Izzi Md. Yusoff, Riza Atiq O. K. Rahmat, Muhamad Nazri Borhan, Shaban Ismael Albrka, Che Husna Azhari, Mohamed Rehan Karim

**Affiliations:** 1 Sustainable Urban Transport Research Centre (SUTRA), Department of Civil and Structural Engineering, Faculty of Engineering and Built Environment, Universiti Kebangsaan Malaysia, UKM, Bangi, Selangor, Malaysia; 2 Department of Mechanical and Materials Engineering, Faculty of Engineering and Built Environment, Universiti Kebangsaan Malaysia, UKM, Bangi, Selangor, Malaysia; 3 Department of Civil Engineering, University of Malaya, Jalan Universiti, Kuala Lumpur, Malaysia; Beihang University, CHINA

## Abstract

Road distress results in high maintenance costs. However, increased understandings of asphalt behaviour and properties coupled with technological developments have allowed paving technologists to examine the benefits of introducing additives and modifiers. As a result, polymers have become extremely popular as modifiers to improve the performance of the asphalt mix. This study investigates the performance characteristics of epoxidized natural rubber (ENR)-modified hot-mix asphalt. Tests were conducted using ENR–asphalt mixes prepared using the wet process. Mechanical testing on the ENR–asphalt mixes showed that the resilient modulus of the mixes was greatly affected by testing temperature and frequency. On the other hand, although rutting performance decreased at high temperatures because of the increased elasticity of the ENR–asphalt mixes, fatigue performance improved at intermediate temperatures as compared to the base mix. However, durability tests indicated that the ENR–asphalt mixes were slightly susceptible to the presence of moisture. In conclusion, the performance of asphalt pavement can be enhanced by incorporating ENR as a modifier to counter major road distress.

## Introduction

Asphalt is one of the most widely used materials in road construction because of its mechanical, viscoelastic, adhesive, and waterproofing properties [1]. The largest use of asphalt is in the production of hot-mix asphalt (HMA), which is primarily used in the construction of flexible pavements. Durable pavements with long service life and low maintenance and rehabilitation costs are preferred; hence, the demand for high-quality asphalt is growing. Consequently, new materials with increased performance such as polymer-modified asphalt (PMA) are being researched and developed. On the other hand, the need for suitable testing methods that can forecast the performance of materials not only during laying but also over the entire working life of the pavement is becoming increasingly important.

Over the past decade, numerous studies have been conducted to modify asphalt and asphalt mixes [2]. Potentially, polymer-modified asphalt (PMA) can significantly improve the performance of HMAs and substantially increase the service life of highway surfaces [2]. Specifically, the addition of polymers significantly improves various properties of asphalt such as elasticity, cohesion, stiffness, and adhesion. This leads to a substantial improvement in the performance and quality of asphalt pavement containing HMA, making the pavement more stable at warmer temperatures and more flexible at colder temperatures. In addition to rutting resistance, a premium polymer can provide a degree of flexibility or elasticity to HMA, thereby improving the fatigue and thermal cracking characteristics of the asphalt mix.

One of the most well-known and widely used categories of polymers is thermoplastic elastomers (TEs). TEs are polymers that exhibit both thermoplastic and elastomeric properties [3]. These polymers derive their strength and elasticity from the physical cross-linking of molecules within a three-dimensional network. Styrene–butadiene–styrene (SBS), the most commonly known TE, can improve the rheological properties of asphalt [4]. Furthermore, SBS increases binder elasticity at high temperatures and improves flexibility at low temperatures. This improvement in turn leads to increased resistance to asphalt rutting at high temperatures and decreased cracking at low temperatures [5]. Styrene–butadiene–rubber (SBR) is another example of an elastomer polymer that increases the ductility of asphalt pavements, which thus becomes more flexible and crack-resistant at low temperatures [6]. Other types of TE additives have also been used as modifiers, such as styrene isoprene styrene (SIS), styrene ethylene butadiene styrene (SEBS), ethylene-propylene-diene-terpolymer (EPDM), isobutene-isoprene-copolymer (IIP), crumb rubber, polybutadiene (PBD), polyisoprene, and natural rubber (NR) [2, 7]. However, it has been proved that the three major types of styrenic block copolymers (SBCs)—SBS, SIS, and hydrogenated styrenic block copolymers (HSBCs)—have the best modifying potential when blended with asphalt [8–10].

In some cases, vulcanized rubber has been used, e.g., crumb rubber. However, obtaining a uniform dispersion within the asphalt poses severe difficulties. Non-uniform dispersion requires high temperatures and long mixing times and can yield a heterogeneous binder, with the rubber acting mainly as a flexible filler [2]. However, crumb rubber is essentially a combination of natural rubber, which improves elasticity, carbon black, and synthetic rubber, both of which improve thermal stability. In addition, crumb rubber has been found to increase rutting resistance and decrease reflective cracking [11]. Nevertheless, it was found that natural rubber showed superior reactivity as compared to crumb rubber and that the reacted particles became tacky, which improved adhesion [12]. The strong cohesion between aggregates is one of the benefits of using natural rubber to modify asphalt [13]. Natural rubber increases the stiffness of the binder at high temperatures, thereby enhancing the latter’s performance, but it renders the binder brittle at low temperatures [14]. Remarkably, natural rubber displays high mechanical strength, outstanding resilience, and excellent elasticity. However, it is also known to exhibit poor wet grip properties and poor weather resistance [15].

The potential of using epoxidized natural rubber (ENR) as a modifier was discovered in the 1980s [15]. ENR is a chemically modified natural rubber created by reacting natural rubber with proxy formic acid [15]. This material exhibits good mechanical properties, offers high strength owing to its ability to bear strain crystallization, and has a high glass transition temperature. These properties in turn facilitate increased oil resistance, enhanced adhesion, damping, and reduced gas permeation [16, 17]. Previous studies also report that ENR increased the viscosity and stiffness and decreased the temperature susceptibility of the binder [18–20].

This study presents an experimental performance evaluation of a base asphalt mix (HMA-0) and an asphalt mix modified with 3% (HMA-3), 6% (HMA-6), 9% (HMA-9), and 12% (HMA-12) ENR (as a percentage of asphalt by weight) in terms of indirect tensile resilient modulus, dynamic creep, rutting, fatigue, and its susceptibility to moisture.

## Materials and methods

### Materials

The asphalt used in this study was 80/100 penetration grade supplied by the asphalt factory at Port Klang, Malaysia. The ENR was obtained from the Malaysian Rubber Board under the trade name of ENR 50, with 53% epoxidation. It was passed through a 2.36 mm mesh sieve (before shearing). The physical properties of the asphalt and ENR used are shown in Table 1. The aggregate used for preparing the mixed samples was obtained from Negeri Roadstone Sdn. Bhd., Malaysia.

**Table 1:** Materials and properties of base Asphalt and ENR.

Materials	Properties	Value	Specification
Asphalt 80/100	Specific Gravity	1.03	ASTM D70
	Penetration @ 25°C	82	ASTM D5
	Softening point (°C)	45.7	ASTM D36
	Viscosity @ 80°C (Pa s)	12.6	ASTM D4402
	Ductility (cm) @ 10°C and 5 cm/min	20	ASTM D113
	Ductility (cm) @ 25°C and 5 cm/min	>100	
ENR	Size (before shearing)	2.36 mm	-
	Specific gravity	0.94	-

### Preparation of the binder

ENR-modified asphalts were produced by mixing 3, 6, 9, and 12% ENR (by weight of asphalt) with the base asphalt using a high shear mixer at 160°C (±1°C) under 4000 rpm for 60, 62, 66, and 66 min, respectively [21]. ENR was added to the asphalt after temperature stabilization at 160°C.

### Asphalt mix preparation

The asphalt mix design was based on the Superpave volumetric mix design. The design equivalent single-axle loads (ESALs) for this study were assumed to be less than 10^7^. This categorises the design in the 3 × 10^6^ to 1 × 10^7^ ESALs (80 KN/ESAL) category or traffic level 4 [22]. Traffic levels are used to determine design requirements, such as the number of design gyrations for compaction, the physical properties of the aggregate, and the volumetric requirements of the mix. The traffic level also determines the level of mix design required. For traffic levels of less than 10^7^ ESALs, a level 2 mix design is recommended [22]. The mix in this study had a nominal maximum particle size aggregate of 19.0 mm. However, six stockpiles of the aggregate consisted of three coarse and three fine aggregates.

### Asphalt mix performance tests

Performance tests were used to relate the laboratory mix design to actual field performance. The following section focuses on laboratory tests done on asphalt mixes.

#### Indirect tensile resilient modulus test

Sample preparation and testing procedures for the indirect tensile resilient modulus test were based on the ASTM-D4123 [23] standard and were carried out using a universal testing machine (UTM) (S1 Fig). The resilient modulus (*M*_R_) is one of the most fundamental and important parameters used among researchers to determine the mechanical properties of asphalt mixes, which is then used in the mechanistic design of pavement structures. It is a non-destructive test and can be defined as the ratio of applied stress to recoverable strain at a particular temperature for a given load.

In this study, three replicate samples (100 mm in diameter and 63.5 ± 2.5 mm in height) for each type of mix were compacted at 4% air voids using a gyratory compactor. Prior to testing, all the samples and test equipment were conditioned at 5, 25, and 40°C in an environmental chamber for a minimum of 2 h. The preconditioned samples were set with a haversine wave pattern with five conditioning pulses followed by five loading pulses during testing, after which the data were recorded.

In addition, the pulse width of the load was set at 100 ms with a pulse repetition period ranging from 300 to 1000 ms. With respect to the pavement, Tayfur et al. [24] suggested a pulse repetition period of 1000, 2000, and 3000 ms in order to simulate high, intermediate, and low traffic volume, respectively.

#### Dynamic creep test

The dynamic creep test, which can also be performed using the UTM (S2 Fig), determines the permanent deformation of the asphalt mix in a laboratory setting. In this study, the dynamic creep test under a haversine load pulse was conducted for the base asphalt mix and the ENR-modified mixes according to the protocols developed by the NCHRP 9–19 Superpave Model [25]. Each category of ENR mix consisted of three replicate samples, which were compacted at 4% air voids, with a diameter of 100 mm and a height of 150 mm. The samples were preconditioned in the environmental chamber at 40°C for at least 4 h before testing commenced. During the initial stages of testing, samples were pre-loaded with a conditioning stress of 10 kPa for 120 s to ensure the platen was flat against the sample. Subsequently, a haversine wave load cycle was applied, which consisted of a 100 kPa stress pulse with a 100 ms pulse width, followed by a 900 ms rest period. The test was terminated when the accumulative strain reached 10,000 micro-strains or until 10,000 cycles were completed, whichever came first.

#### Rutting test

This test simulates wheel passes on pavement. The procedure followed is as described in BS-598-110 [26]. A Wessex wheel-tracking device was used to perform this test on a laboratory scale. Wheel-tracking test samples were produced for each mix once the respective design aggregate and optimum binder content (OBC) was known (S3 Fig). In this study, one mould was used to hold the rut sample in the wheel-tracking machine. The height of the mould exactly matched the original slab mould of the Wessex wheel-tracking device. Approximately 3,700 gm of mix was compacted to 7 ± 0.5% air voids to obtain a sample with an area of 300 × 300 mm and a height of 65 ± 1 mm. The samples were allowed to cool for 24 h at room temperature after compaction before calculating the air voids content as per test requirements. The rut test was conducted at 50°C. Before testing, the samples were conditioned for at least 4 h at the test temperature. The test was conducted only in dry conditions. The samples were subjected to simulated traffic with a simple harmonic motion by applying 520 N loads for 1 h.

#### Flexural fatigue test

The flexural fatigue test is conducted to evaluate the fatigue characteristics of the mixes. However, fatigue cracking of pavement is considered to be more of a structural problem than simply a material problem. A repeated load flexure testing machine was developed to perform controlled strain flexure fatigue tests on asphalt mix beams. Symmetrical four-point loading produced a constant bending moment over the middle third of a 375-mm-long beam sample that was 50 mm deep and 62.5 mm wide (S4 Fig). The test was conducted in accordance with AASHTO [27]. Particularly, repeated haversine loads were applied to the third point of the sample, which was placed inside the environmental chamber to maintain the temperature at 20°C during the test. In the constant strain beam fatigue test, a constant strain was maintained on the sample, but the magnitude of the load was allowed to decrease with increasing load cycles. With this method, the samples did not break during the test. Instead, failure was defined as 50% loss in initial beam stiffness.

#### Moisture susceptibility

It is very important to evaluate the moisture sensitivity of the design mix. This step is accomplished by performing the AASHTO method on the design aggregate structure to determine the design asphalt binder content [28]. Base asphalt and ENR-modified asphalt samples with diameters of 100 mm and heights of 63.5 ± 2.5 mm were compacted to approximately 7% air voids. One subset of the three samples was defined as the control subset and the rest were defined as the conditioned subset. The conditioned subset was subjected to partial vacuum saturation followed by an optional freeze cycle and a 24 h thaw cycle at 60°C. All samples were tested to determine their indirect tensile strength. The moisture sensitivity was defined as the ratio of the tensile strength of the conditioned subset to the tensile strength of the dry (control) subset. The minimum criteria for tensile strength ratio was 80% [28].

## Results and discussion

Performance testing is an area of Superpave that has yet to be implemented widely. The performance tests discussed in this study have been used by various researchers. However, as with asphalt binder characterisation, the challenge in HMA performance testing is to develop physical tests that can satisfactorily characterise key HMA performance parameters and determine how these parameters will change throughout the life of a pavement. The key parameters to be considered are deformation resistance, fatigue life, stiffness, and moisture susceptibility [29]. However, the physical characterisations of the asphalt mix are described in this study. These descriptions are based on typical laboratory characterisation procedures. The effect of 3, 6, 9, and 12% ENR content (as a percentage of the base asphalt by weight) on the mechanical performance of the modified mixes was investigated and compared to that of HMA-0.

### The design aggregate structure

The nominal maximum particle size was chosen to be 19.0 mm. However, the six stockpiles of materials consisted of three coarse and three fine particle sizes. Aggregates were washed, oven dried and sieved to separate sizes before adding them to the mix to ensure that the gradation of the Superpave mix design was strictly followed. The following sieve sizes were used in the gradation: 19.00, 9.50, 4.75, 2.36, 0.30, and 0.075 mm sieves.

To begin with, the bulk specific gravity (*G*_sb_) of the coarse and fine aggregates was calculated for all sieve sizes and the results are shown in Table 2. The apparent specific gravity (*G*_sa_) and effective specific gravity (*G*_se_) were also determined from these tests. Further analysis of the Superpave system consensus and source aggregate properties was carried out as this was critical to achieving high-performance HMA pavement. The results confirmed that the aggregates from the quarry complied with the standard specification criteria. Table 2 shows the results of the aggregate property tests.

**Table 2:** Aggregate property test results.

Aggregate properties	Results	Criteria	Standard
*G*_sb_ of coarse aggregate	2.58	-	ASTM C 127
*G*_sb_ of fine aggregate	2.61	-	ASTM C 128
Flakiness (%)	6.00	<20	BS 812: section 105.1: 1989
Fine aggregate angularity (%)	51.5	>45	AASHTO T33
Elongation (%)	16.0	<20	BS 812
Sand equivalent test	48.5	>45	AASHTO 176
Los Angeles test (%)	32.13	<45	ASTM C: 131–81
Soundness test (%)	6.1	<12	ASTM C88
Deleterious materials (%)	0.5	0.2 to 10	ASTM C142

**Fig 1:**
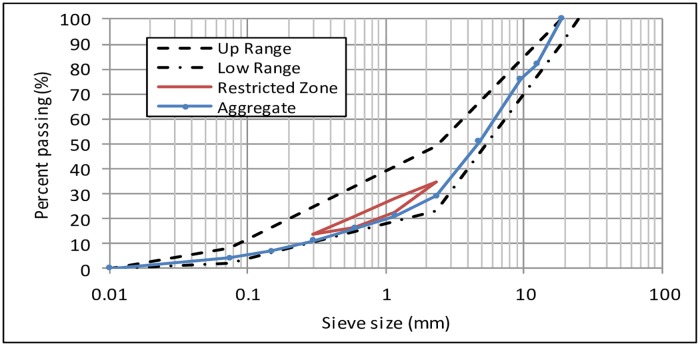
Aggregate gradation.

In general, the aggregate fulfilled the Superpave mix design requirements and was found suitable for use as a pavement material. Fig 1 shows the aggregate gradation used in this study. Aggregate gradation was prepared using 19.0 mm as the nominal maximum size in order to comply with the Superpave gradation limits.

### Binder properties

Material characterisation based on standard and Superpave tests is presented in Table 3 [1, 30]. These results complied with the specifications set by the standard criteria under binder PG 76.

**Table 3:** Binder properties.

		ENR content (by % weight of asphalt)				
		ENRMA-0	ENRMA-3	ENRMA-6	ENRMA-9	ENRMA-12
	**Binder description (and criteria)**	0%	3%	6%	9%	12%
Original binder	Penetration at 25°C, 1/10 mm	82	73	64	52	53
	Softening point, °C	45.70	50.60	53.80	56.80	57.00
	Viscosity @ 135°C (max. 3 Pa s), Pa s	0.244	0.469	0.606	0.787	0.694
	Flash point (min. 230°C), °C	275	308	329	353	371
	Dynamic shear at 10 rad/s (G*/sinδ), (min. 1 kPa), kPa	0.177	1.382	2.103	1.410	1.680
RTFOT	RTFOT weight loss% (max. 1%)	0.36	0.31	0.29	0.26	0.25
	Dynamic shear at 10 rad/s (G*/sinδ), (min. 2.20 kPa), kPa	0.969	2.753	3.826	3.789	3.480
PAV	Dynamic shear at 10 rad/s (G*/sinδ), (max. 5000 kPa), kPa	3962	2291	2414	880	1036

### Volumetric properties of mixes

Asphalt mixes were successfully developed in accordance with the procedures described in the Superpave mix design standard [22]. In this study, mixes with five different asphalt contents (4, 4.5, 5, 5.5, and 6%) were prepared (N = 3 for all the samples) for each base and modified HMA. Specimens were prepared by blending mineral aggregates in increments of 0.5% binder (i.e., 4.0, 4.5, 5.0, 5.5 and 6.0%) by weight of aggregate for the base and modified HMA.

The optimum binder content (OBC), which ensured acceptable volumetric properties as compared to the established mix criteria, was based on the Superpave gyratory compactor (SGC) specimens with 4% air voids. The volumetric properties consisted of OBC, effective binder content (*P*_be_), voids in mineral aggregate (VMA), voids filled with asphalt (VFA), and the ratio of dust to the effective binder content (*P*0.075/*P*_be_). The major factors that determine the stability and durability of Superpave HMA mixes are VMA, VFA, air voids, and dust proportion. Table 4 summarises the volumetric properties of the design mixes corresponding to the OBC of the mix, along with mix design criteria. The results for all the mixes revealed that their properties satisfied all the criteria set by the Superpave system.

**Table 4:** Volumetric properties of all mixes.

Mix Properties	HMA-0	HMA-3	HMA-6	HMA-9	HMA-12	Criteria
Binder type	ENRMA-0	ENRMA-3	ENRMA-6	ENRMA-9	ENRMA-12	
OBC (%)	4.86	4.96	5.08	5.31	5.87	-
Air Voids (%)	4.00	4.00	4.29	4.54	4.69	3–5
VMA (%)	14.44	15.31	17.44	18.99	19.15	≥13
VFA (%)	74.14	74.87	75.00	75.00	75.00	65–75

### Resilient modulus test results

Resilient modulus is an important variable used to measure pavement response in terms of dynamic stresses corresponding to strains. However, this test can be used to evaluate low-temperature cracking, fatigue, and permanent deformation of asphalt mixes [31–33]. When elastic-layered system theory is used to design the asphalt mix, the modulus of asphalt is considered a basic design parameter [34].

During testing, horizontal deformation is measured from both sides of the specimen and the resilient modulus is calculated accordingly. In this test, the base and modified HMA at 5, 25, and 40°C and at 0.33, 0.5, and 1 Hz were analysed, compared, and characterised. In general, it was observed that the resilient modulus for all mixes increased inversely with temperature, regardless of the availability of the modifier. In contrast, some studies have reported a decrease in the resilient modulus; however, numerous polymers were used as modifiers [24].

**Fig 2:**
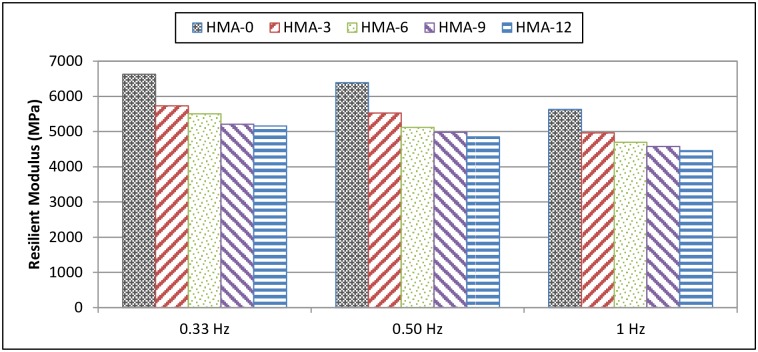
Resilient modulus of HMA mixes at 5°C.

**Fig 3:**
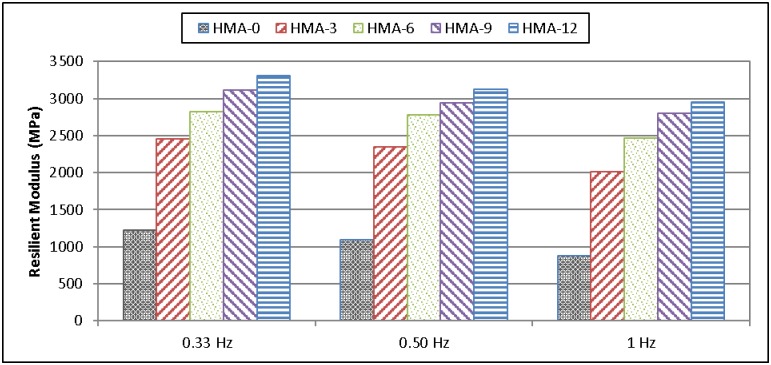
Resilient modulus of HMA mixes at 25°C.

**Fig 4:**
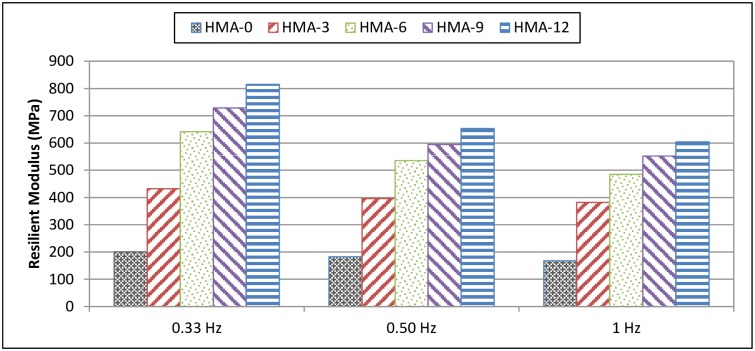
Resilient modulus of HMA mixes at 40°C.

At low temperature (5°C), the resilient modulus of the base HMA-0 mix was higher than that of the modified mixes. This indicates that the elasticity of the mix decreased after the addition of the polymer modifier, as shown in Fig 2. On the other hand, as the pulse repetition frequency during loading was increased from 0.333 Hz to 1 Hz, the resilient modulus values decreased. However, at 5°C, HMA-0 showed the highest resilient modulus for low-frequency loading. At 25 and 40°C, the resilient modulus values increased with increasing ENR content. This indicates that the elasticity increased after adding the polymer modifier, as shown in Figs 3 and 4.

Significantly, for all temperatures, the resilient modulus decreased with increasing loading frequency. Finally, these results indicated that HMA-0 was more susceptible to cracking at low temperature because of the increase in the resilient modulus and to fatigue cracking and permanent deformation at intermediate and high temperatures, respectively, because of the decrease in the resilient modulus.

Tayfur et al. [24] modified various types of polymers and reported that the base asphalt had a higher resilient modulus as compared to PMA mixes at 5°C, which meant that the base asphalt had a higher elasticity modulus (stiffness) and hence a lowest cracking resistance. Loading frequency increased the resilient modulus, especially at 25°C and 40°C. Finally, the results indicated that the resilient modulus values of the base mixes, especially at 5°C, were higher than those of the PMA mixes, but at higher temperatures (25°C, 40°C), the values tended to decrease.

### Dynamic creep test results

**Fig 5:**
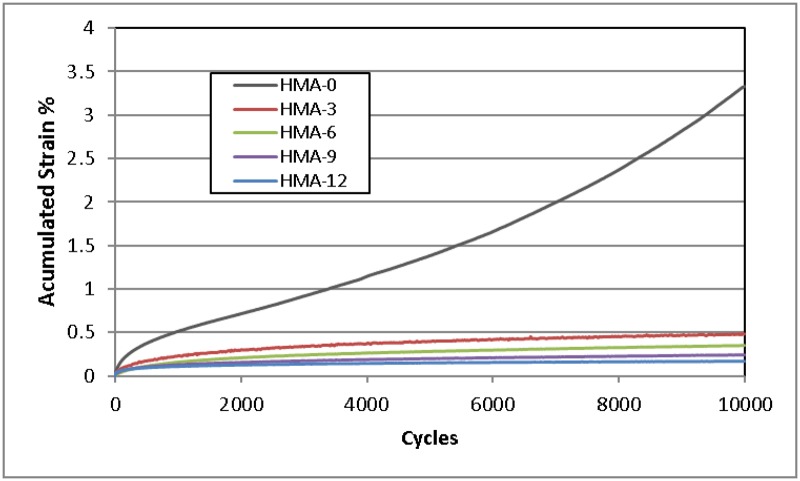
Dynamic creep curves for all asphalt mixes.

The dynamic creep test is used to determine the strength of HMA mixes to plastic deformation. Fig 5 shows the comparison between the accumulated strain of HMA-0 and the modified mixes at 40°C and a stress of 100 kPa. The dynamic creep curve consists of three parts: primary, secondary, and tertiary. The accumulated strain was recorded at each load cycle and the test was terminated when the numbers of cycles reached 10,000. Because the loading period was terminated at 10,000 cycles, not all specimens failed before reaching the maximum number of cycles. Under 10,000 load cycles, all axial strains exhibited a curved relationship with load cycle in the strain versus load cycle plot.

Extreme permanent deformation was observed in the HMA-0 mix as compared to all modified mixes, as shown in Fig 5. This behaviour can be attributed to the influence of ENR on the binder and thus on the mix. The mechanical properties of all modified mixes rely on the properties of the binder at high temperatures, especially for permanent deformation. Therefore, it is expected that the resistance to permanent deformation would increase in the modified mix. Finally, adding ENR to asphalt mixes significantly decreased the asphalt’s susceptibility to permanent deformation.

Similar polymer behaviour has been reported [35]; however, in that case, different percentages of styrene butadiene styrene (SBS) were used as the modifier. Khodaii and Mehrara found that adding SBS to the asphalt increased the asphalt mix’s resistance to permanent deformation [35]. Fernando and Guirguis [36] indicated that the addition of 4% rubber decreases the creep compliance of asphalt mix by 42% at 45°C. Eaton et al. [37] reported that the total creep was higher for the rubber mixes, pointing out the benefits of their performance at lower temperatures, that is, greater elasticity and better resistance to thermal cracking.

### Rutting test results

**Fig 6:**
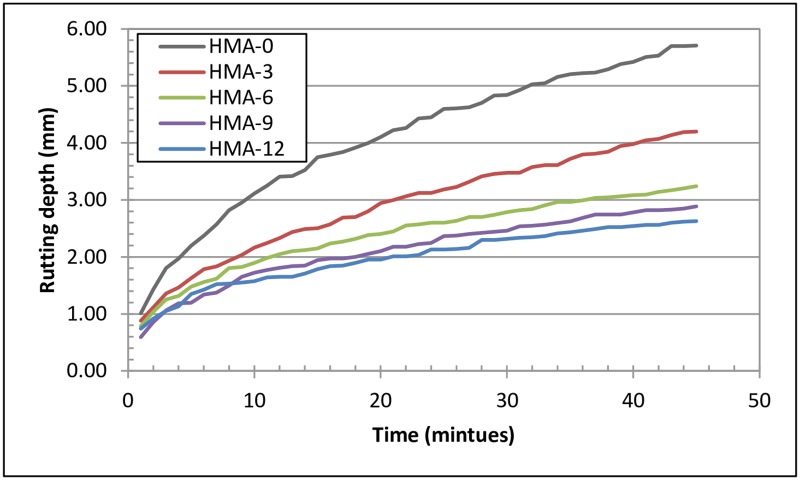
Rutting test results for all asphalt mixes.

The rutting test is a simulative test, also known as the torture test, conducted using a dry wheel-tracking device. A test temperature of 50°C was chosen to simulate extreme environmental conditions for the HMA mixes. The wheel passed over the HMA specimens and was terminated after 45 min. In general, all modified mixes exhibited good rutting resistance as compared to HMA-0, as shown in Fig 6. Although the HMA-12 mix exhibited the lowest rutting value, HMA-0 exhibited the highest rutting value. Clearly, this indicates the high resistance of the modified mixes to rutting as compared to HMA-0.

The performance improvement of a base asphalt mix with addition of SBS has been reported [38], which was mainly attributed to the enhanced resistance to rutting imparted to the pavement by SBS. According to Shih et al. [39], addition of crumb rubber and SBR increased the rutting resistance of asphalt mixes. The results from laboratory study showed that the crumb rubber- modified and SBR-modified asphalt had higher stiffness at 60°C than the base mixes.

### Flexural fatigue test

**Fig 7:**
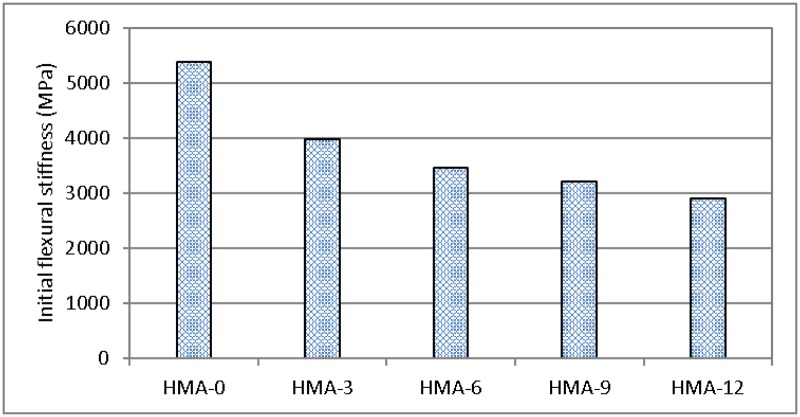
Fatigue results using the initial flexural stiffness for all asphalt mixes.

**Fig 8:**
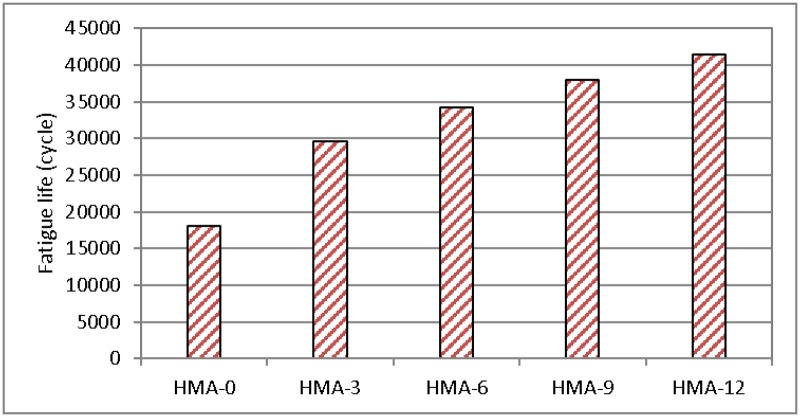
Fatigue results using the number of cycles to failure for all asphalt mixes.

Fig 7 shows the fatigue life and number of cycles to failure, which is defined as the loading cycle when the flexural stiffness of the mix drops to 50% of its initial value. Fig 8 shows the initial flexural stiffness for all the mixes.

It is obvious that HMA-0 predominantly affected the initial stiffness of the mix as compared to the modified mixes, although the results from the fatigue life tests show a different aspect, i.e., HMA-0 failed after 18,030 cycles, whereas HMA-12 failed after 41,453 cycles. This indicates that the fatigue life improved after adding the modifier to the base asphalt mix. On the other hand, a decrease in the initial stiffness of the modified mix indicates a decrease in the elasticity of the mix. A previous study of an asphalt mix that used SBS as a modifier under a strain of 600 με found that SBS also increased the fatigue life of the asphalt mix [40]. Raad and Saboundjian [41] investigated the fatigue behaviour of the different mixes using controlled-strain third-point flexural beam tests. Results indicated that the crumb rubber modified asphalt mixes can enhance their fatigue resistance. The magnitude of improvement appears to depend on the degree and type of rubber modification. Lundy et al. [42] presented the three case histories in which ground rubber modification was used in the construction of asphalt mix. The results showed that the modified material has improved fatigue characteristics compared to the base mix as a result of the thicker asphalt films.

### Moisture susceptibility

**Fig 9:**
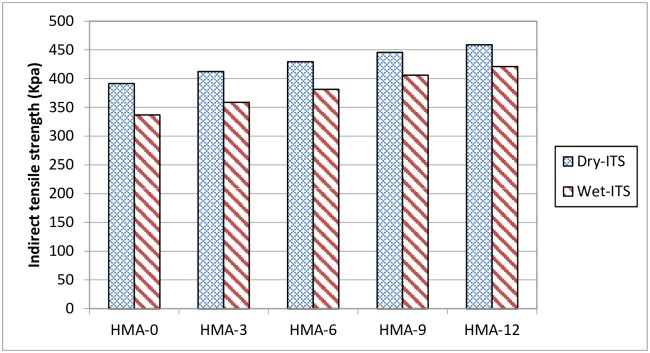
Indirect tensile strength results for all asphalt mixes.

**Fig 10:**
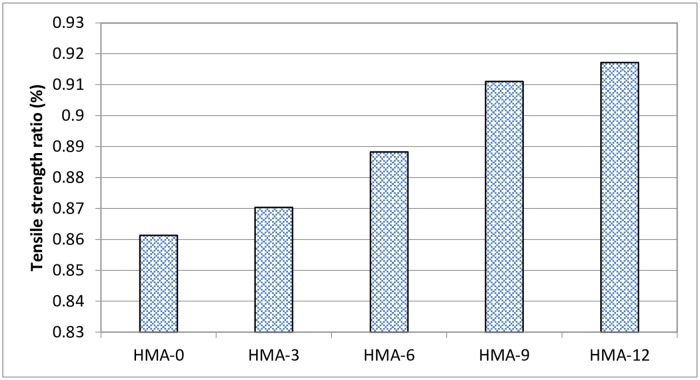
Tensile Strength Ratio (TSR) results for all asphalt mixes.

The indirect tensile strength (ITS) results of each mix under the dry and conditioned cases were averaged based on the results of three specimens [28]. All the ITS results are shown in Figs 9 and 10. All mixes tested were prepared such that their air void content was 7 ± 0.5%. The results showed that the ITS values of all the dry specimens decreased when they were conditioned, implying deterioration in the mixes, which affected the strength of the HMA mix.

The tensile strength ratio (TSR) indicates the susceptibility of the HMA mix to moisture damage. All mixes met the required minimum 80% TSR value as specified in AASHTO T283. The indirect tensile strength test showed that the modified mixes exhibited higher tensile strength as compared to HMA-0 before and after conditioning, which proved that HMA-0 was more susceptible to moisture damage. A previous study that used SBS (HMA-SBS)-modified asphalt also reported a reduction in the susceptibility of HMA to moisture damage [40]. It was found that the SBS-HMA asphalt samples achieved a higher TSR value (88.9%) than the base HMA samples (76.7%). On the other side, it was found that adding Elvaloy to the asphalt mix increased the susceptibility to moisture damage [43]. Coating smooth, rounded, siliceous gravel aggregates with cement plus SBR latex for use in asphalt mix increased the resistance to moisture damage [44].

## Conclusions

The primary objective of this study was to investigate the performance characteristics of ENR-modified asphalt mixes through indirect tensile resilient modulus, dynamic creep, rutting, and fatigue tests and to investigate the durability of the ENR-modified asphalt mixes when exposed to moisture. The most important conclusions derived from this study are as follows:

- In terms of the load-bearing capacity, adding ENR decreased the resilient modulus at low temperatures but increased it at intermediate and high temperatures.
- The permanent deformation of ENR-HMA at 40°C and 50°C was dominated by the ENR content, where ENR-HMA produced less deformation as compared to base-HMA. This is attributed to the presence of highly elastic rubber in HMAs at high temperature.
- The ENR modifier improved the HMA in terms of fatigue life, where ENR-HMA showed greater resistance to fatigue cracking as compared to base-HMA. However, the initial stiffness increased after adding ENR, indicating a decrease in the elasticity of ENR-HMA.
- Moisture damage was reduced after modifying the mixes with ENR, indicating that ENR-HMA was less susceptible to moisture damage as compared to base-HMA. Although this improvement was not significant, the results remain within the standard requirements.

## Supporting information

S1 Fig Indirect tensile resilient modulus test instrument. (TIF)

S2 Fig Dynamic creep test instrument. (TIF)

S3 Fig Rutted sample from wheel tracking test. (TIF)

S4 Fig Flexural fatigue test instrument. (TIF)

## References

[1] Asphalt-Institute. The asphalt handbook. 7th Edition ed. USA: Asphalt Institute; 2007.

[2] Hunter RN, Self A, Read J. The Shell bitumen handbook. 6th ed London: ICE Publishing; 2015.

[3] Ferry JD. Viscoelastic Properties of Polymers. 3rd ed New York: John Wiley and Sons; 1980.

[4] Airey GD. Rheological properties of styrene butadiene styrene polymer modified road bitumens. Fuel. 2003;82(14):1709–19. 10.1016/S0016-2361(03)00146-7.

[5] Lu X, Isacsson U. Rheological characterization of styrene-butadiene-styrene copolymer modified bitumens. Construction and Building Materials. 1997a;11(1):23–32.

[6] Becker Y, Méndez MP, Rodri Y. Polymer modified asphalt. Vis Technol. 2001;9:39–50.

[7] Mo L, Shu D, Li X, Huurman M, Wu S. Experimental investigation of bituminous plug expansion joint materials containing high content of crumb rubber powder and granules. Materials & Design. 2012;37(0):137–43. 10.1016/j.matdes.2012.01.003.

[8] BALaWC Vonk. Thermoplastic rubber/bitumen blends for roof and road. 5th ed London 1984.

[9] Jun L, Yuxia Z, Yuzhen Z. The research of GMA-g-LDPE modified Qinhuangdao bitumen. Construction and Building Materials. 2008;22(6):1067–73. 10.1016/j.conbuildmat.2007.03.007.

[10] Moreno F, Sol M, Martín J, Pérez M, Rubio MC. The effect of crumb rubber modifier on the resistance of asphalt mixes to plastic deformation. Materials & Design. 2013;47(0):274–80. 10.1016/j.matdes.2012.12.022.

[11] Yildirim Y. Polymer modified asphalt binders. Construction and Building Materials. 2007;21(1):66–72. 10.1016/j.conbuildmat.2005.07.007.

[12] Proceedings, first asphalt-rubber user-producer workshop. Asphalt-rubber User-producer Workshop 1980; Scottsdale, Arizona, USA

[13] Michael A. Heitzman PE. State of the practicel design and construction of asphalt paving materials with crumb rubber modifier. 1992 Contract No.: FHWA-SA92-022.

[14] Yousefi AA. Rubber-modified bitumens. Iranian Polymer Journal. 2002;11 (5):303–9.

[15] Yoksan R. Epoxidized natural rubber for adhesive applications. Kasetsart Journal (Natural Science). 2008;42:325–32.

[16] G IR. Epoxidised natural rubber. Journal Nat Rubber. 1991;6(3):184–205.

[17] Ismail H, Hairunezam HM. The effect of a compatibilizer on curing characteristics, mechanical properties and oil resistance of styrene butadiene rubber/epoxidized natural rubber blends. European Polymer Journal. 2001;37(1):39–44. 10.1016/S0014-3057(00)00099-9.

[18] Al-Mansob RA, Ismail A, Alduri AN, Azhari CH, Karim MR, Yusoff NIM. Physical and rheological properties of epoxidized natural rubber modified bitumens. Construction and Building Materials. 2014;63:242–8.

[19] Ismail A, Al-Mansob RA, Yusoff BM, Izzi N, Karim MR. Effect of Toluene as disperser on the Bitumen modified with epoxidized natural rubber with aging simulation. Australian Journal of Basic & Applied Sciences. 2012;6(12).

[20] Al-Mansob RA, Ismail A, Yusoff NIM, Azhari CH, Karim MR, Alduri A, et al Rheological characteristics of epoxidized natural rubber modified bitumen. Applied Mechanics and Materials. 2014;505:174–9.

[21] Al-Mansob RA, Ismail A, Yusoff NIM, Albrka SI, Azhari CH, Karim MR. Rheological characteristics of unaged and aged epoxidised natural rubber modified asphalt. Construction and Building Materials. 2016;102:190–9.

[22] Cominsky RJ, Huber GA, Kennedy TW, Anderson M, Group HR. The Superpave mix design manual for new construction and overlays: Strategic Highway Research Program Washington, DC; 1994.

[23] ASTM-D4123. American Society for Testing and Materials. Standard test method for indirect tension test for resilient modulus of bituminous mixtures ASTM D4123. Conshohocken, Pennsylvania, USA1995.

[24] Tayfur S, Ozen H, Aksoy A. Investigation of rutting performance of asphalt mixtures containing polymer modifiers. Construction and Building Materials. 2007;21(2):328–37. 10.1016/j.conbuildmat.2005.08.014.

[25] Witczak MW. Simple performance tests: summary of recommended methods and database.Transportation Research Board; 2005.

[26] BS-598-110. Sampling and examination of bituminous mixtures for roads and other paved areas. Methods of test for the determination of wheel-tracking rate and depth. British Standard, 1998.

[27] AASHTO-T321. Determining the fatigue life of compacted hot mix asphalt (HMA) subjected to repeated flexural bending. American Association of State Highway & Transportation Officials Washington DC. 2007.

[28] AASHTO-T283. Resistance of compacted asphalt mixtures to moisture-induced damage. American Association of State Highway & Transportation Officials Washington DC. 2003.

[29] Brown ER, Kandhal PS, Roberts FL, Kim YR, Lee D-Y, Kennedy TW, et al Hot mix asphalt materials, mixture design, and construction. 3rd Edition ed Lanham, Maryland: NAPA Research and Education Foundation; 2009.

[30] Christensen DW, Anderson DA. Interpretation of dynamic mechanical test data for paving grade asphalt cements. Journal of the Association of Asphalt Paving Technologists. 1992;61:67–116.

[31] Kulash DJ, editor Toward performance–based specifications for bitumen and asphalt mixtures. Proceedings of the ICE-Transport. 1994.

[32] Tian P, Zaman MM, Joakim G, Laguros G. Variation of resiliency modulus of aggregate base and its influence on pavement performance. Journal of Testing and Evaluation. 1998;26(4):329–35.

[33] Brown E, Foo KY. Evaluation of variability in resilient modulus test results (ASTM D 41 23). 1989.

[34] Al-Hadidy AI, Tan Y-q. Mechanistic analysis of ST and SBS-modified flexible pavements. Construction and Building Materials. 2009;23(8):2941–50. 10.1016/j.conbuildmat.2009.02.023.

[35] Khodaii A, Mehrara A. Evaluation of permanent deformation of unmodified and SBS modified asphalt mixtures using dynamic creep test. Construction and Building Materials. 2009;23(7):2586–92.

[36] Fernando M, Guirguis H. Natural rubber for improved surfacing. Australian Road Research. 1984;12(2).

[37] Eaton RA, Roberts RJ, Blackburn RR. Use of scrap rubber in asphalt pavement surfaces. DTIC Document, 1991.

[38] Sargand S, Kim S, editors. Performance evaluation of polymer modified and unmodified Superpave mixes. Second International Symposium on Maintenance and Rehabilitation of Pavements and Technological Control Segundo Simposio Sobre Manutencao e Rehabilitacao de Pavimentos e Controle Technologico; 2001.

[39] Shih C-T, Tia M, Ruth BE. Evaluation of the effects of crumb rubber and SBR on rutting resistance of asphalt concrete. Preprints of Papers-American Chemical Society Division Fuel Chemistry. 1996;41:1227–31.

[40] Bennert T, Martin J-V. Polyphosphoric acid in combination with Styrene-Butadiene-Styrene block copolymer—laboratory mixture evaluation. Asphalt Paving Technology-Proceedings Association of Asphalt Technologists. 2010;79:773.

[41] Raad L, Saboundjian S. Fatigue behavior of rubber-modified pavements. Transportation Research Record: Journal of the Transportation Research Board. 1998;(1639):73–82.

[42] Lundy JR, Hicks R, Zhou H. Ground rubber tires in asphalt-concrete mixtures—three case histories. Use of waste materials in hot-mix asphalt: ASTM International; 1993.

[43] Witczak MW, Hafez I QX. Laboratory characterization of Elvaloy modified asphalt mixtures. Maryland: College Park,: University of Maryland, 1995.

[44] Kim M-G, Button JW, Park D-W. Coatings to improve low-quality local aggregates for hot mix asphalt pavements. 1999.

