# Supplementary figures and images for: Engineering characterisation of epoxidized natural rubber-modified hot-mix asphalt
Source: PLoS One. 2017 Feb 9;12(2):e0171648. doi: 10.1371/journal.pone.0171648 (PMC5300135; doi:10.1371/journal.pone.0171648)

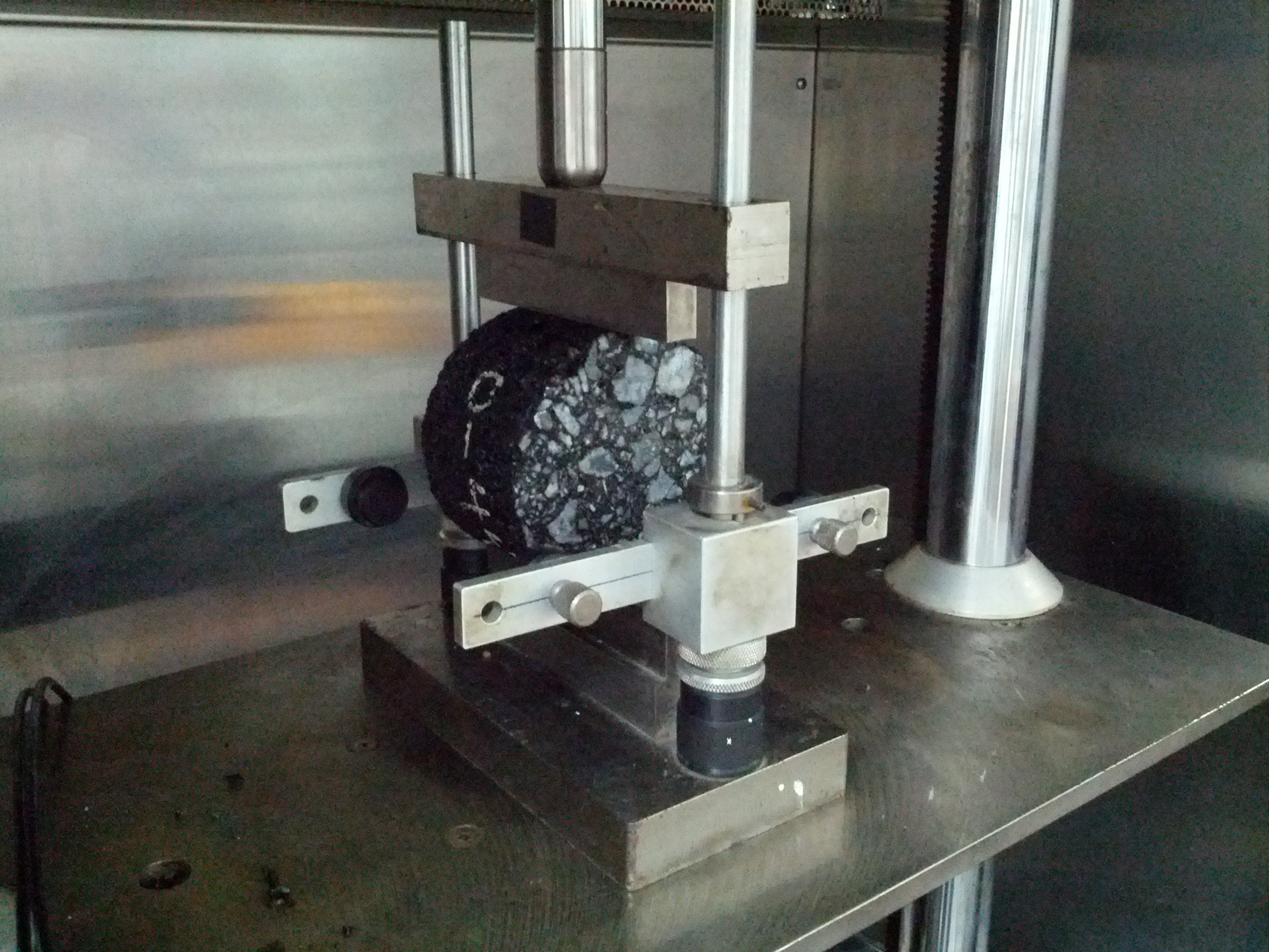

Supplement: S1 Fig — (TIF) [file pone.0171648.s001.tif]

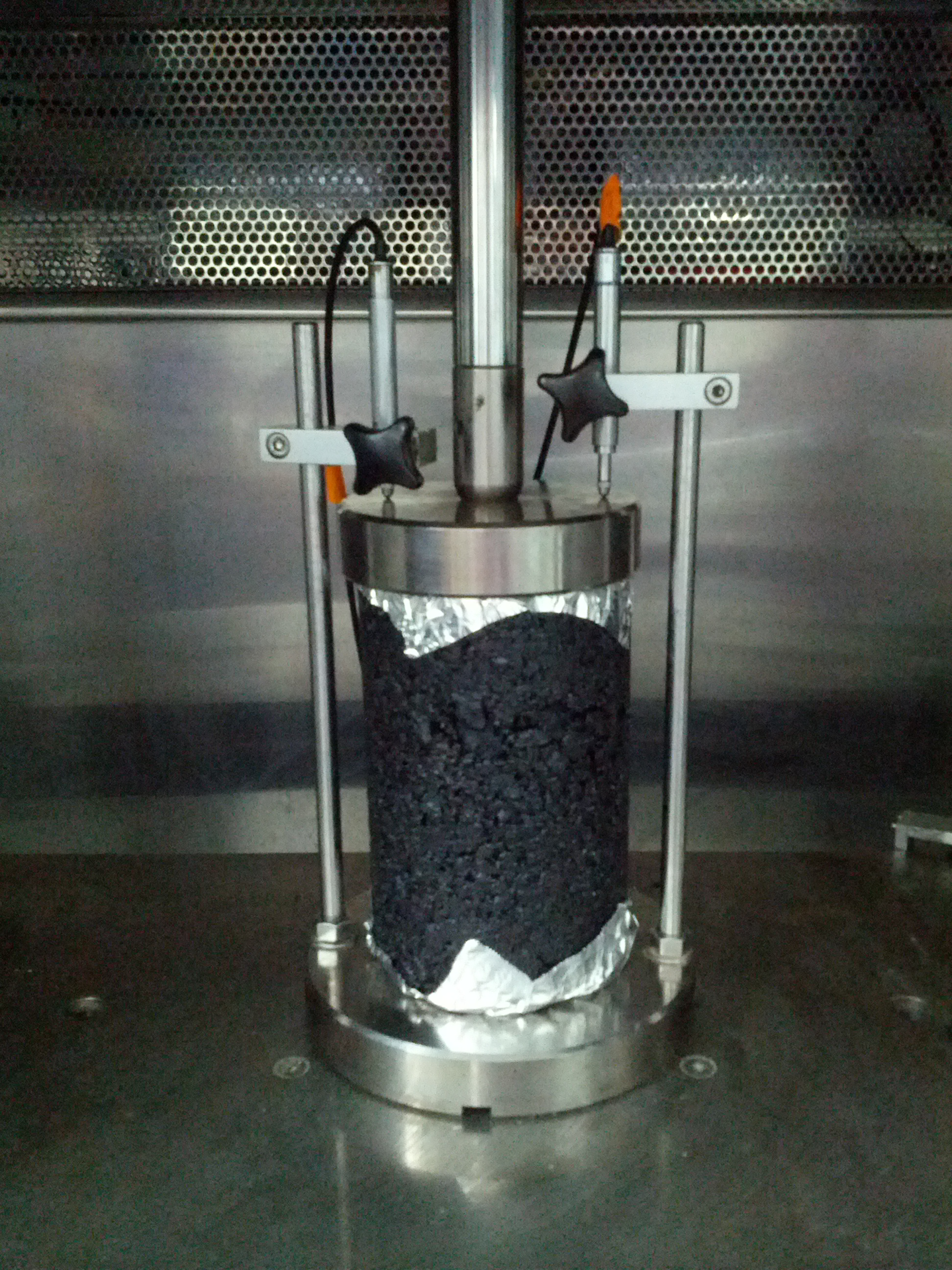

Supplement: S2 Fig — (TIF) [file pone.0171648.s002.tif]

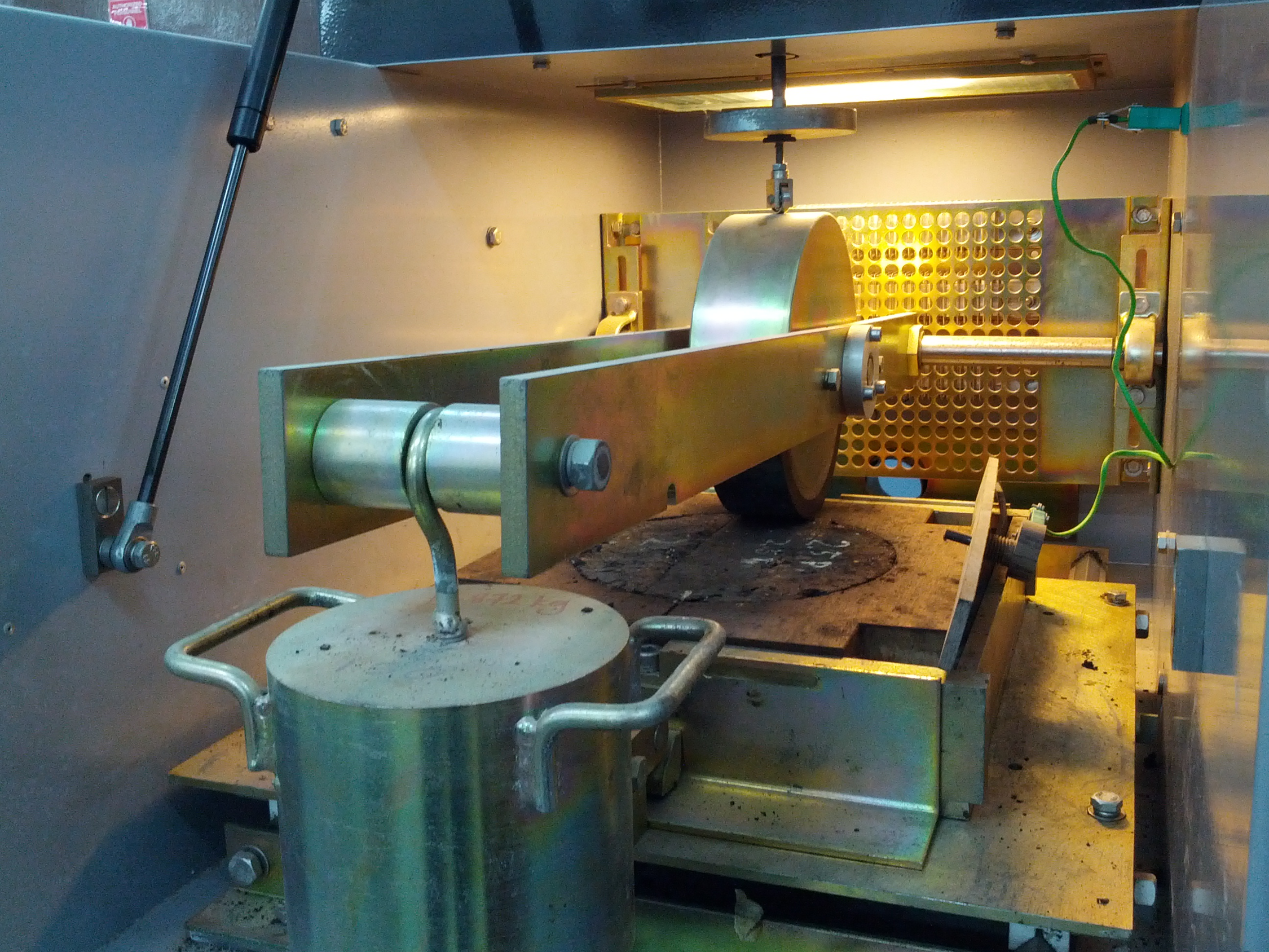

Supplement: S3 Fig — (TIF) [file pone.0171648.s003.tif]

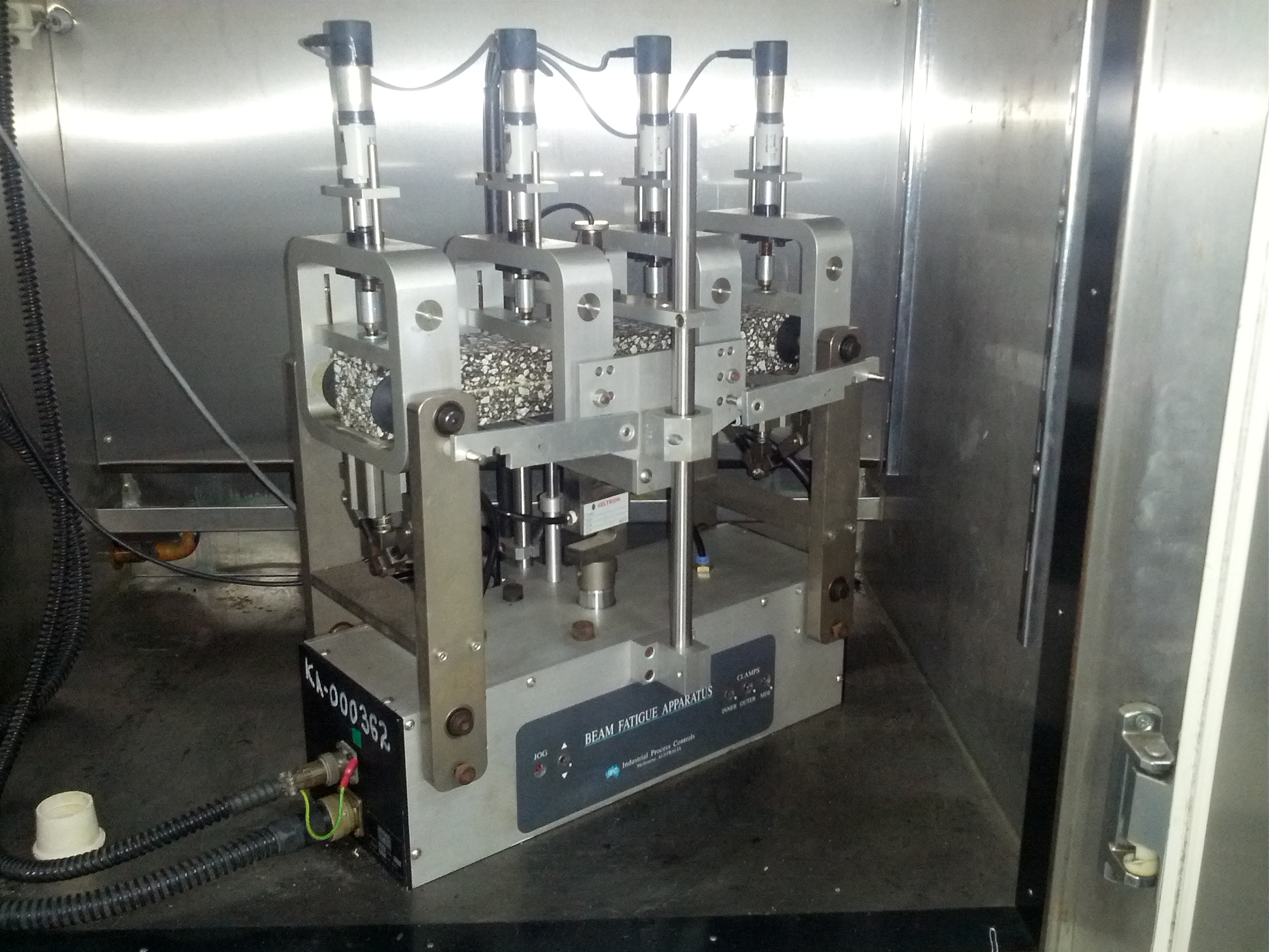

Supplement: S4 Fig — (TIF) [file pone.0171648.s004.tif]
